# Temporomandibular Myofascial Pain Syndrome—Aetiology and Biopsychosocial Modulation. A Narrative Review

**DOI:** 10.3390/ijerph18157807

**Published:** 2021-07-23

**Authors:** Paulina Golanska, Klara Saczuk, Monika Domarecka, Joanna Kuć, Monika Lukomska-Szymanska

**Affiliations:** 1 Department of General Dentistry, Medical University of Lodz, 251 Pomorska St., 92-213 Lodz, Poland; paulina.golanska@umed.lodz.pl (P.G.); klara.saczuk@umed.lodz.pl (K.S.); monika.domarecka@umed.lodz.pl (M.D.); 2 Department of Prosthodontics, Medical University of Bialystok, 24 A M. Sklodowskiej-Curie St., 15-276 Bialystok, Poland; joanna.kuc@umb.edu.pl

**Keywords:** temporomandibular myofascial pain syndrome, biopsychosocial profile modulation, limbic system, stress, bruxism

## Abstract

This review elaborates on the aetiology, diagnosis, and treatment of temporomandibular (TMD) myofascial pain syndrome (MPS) regulated by psychosocial factors. MPS impairs functioning in society due to the accompanying pain. Directed and self-directed biopsychosocial profile modulation may be beneficial in the treatment of MPS. Moreover, nutrition is also a considerable part of musculoskeletal system health. A fruit and vegetable diet contributes to a reduction in chronic pain intensity because of its anti-inflammatory influence. Cannabidiol (CBD) oils may also be used in the treatment as they reduce stress and anxiety. A promising alternative treatment may be craniosacral therapy which uses gentle fascia palpation techniques to decrease sympathetic arousal by regulating body rhythms and release fascial restrictions between the cranium and sacrum. MPS is affected by the combined action of the limbic, autonomic, endocrine, somatic, nociceptive, and immune systems. Therefore, the treatment of MPS should be deliberated holistically as it is a complex disorder.

## 1. Introduction

Myofascial pain syndrome (MPS) constitutes one of the most important health chronic problems which also occurs in clinical dental practice. MPS is defined as a common pain disorder in which the development of trigger points (TPs) is observed [1]. It can occur as a local or global dysfunction. As a particular type of temporomandibular disorder (TMD) this condition is demonstrated by the pain of muscle origin as well as pain spreading beyond the boundary of the masticatory muscles. Secondary to pain, limitation in mandibular movement could be observed [2]. Additionally, myofascial TP is specified as a hyperirritable spot that arises from muscles and their connective tissue [3]. These taut bundles are painful when pressured, stretched, overstrained, or contracted, and usually have a clear pattern of referred pain. Clinically, there exist active and latent TPs [4].

It is worth mentioning, MPS affects a majority of the general population and impairs mobility and concurrent pain is a critical factor for patients as it reduces the overall sense of well-being and hinders functioning in society.

The aim of the current review was to provide a comprehensive review on the aetiology, diagnosis, and treatment of temporomandibular myofascial pain syndrome.

## 2. Search Strategy

The review complies with the recommendations of the Scale for the Assessment of Narrative Review Articles (SANRA) [5]. An electronic search was carried out in the SCOPUS and PubMed digital databases using the keywords related to the topic search and combining the keywords using “AND” and “OR”. The search strategy employed was as follows: (Myofascial pain syndrome) AND (trigger points); (Myofascial pain syndrome) AND (Relaxation techniques); (Myofascial pain syndrome) AND (meditation); (Myofascial pain syndrome) AND (Treatment); (Myofascial pain syndrome) AND (Occurrence); (Myofascial pain syndrome) AND (Statistics); (Myofascial pain syndrome) AND (Limbic system); (Myofascial pain syndrome) AND (Diagnosis); (Bruxism) AND (Myofascial pain syndrome); (Biopsychosocial profile modulation); (Cognitive behavioural therapy); (Biofeedback); (Sleep hygiene measures); (Meditation); (Schultz autogenic training); (Edmund Jacobson’s progressive muscle relaxation); (Diaphragmatic breathing); (Craniosacral therapy).

The review was extended to the articles from their references and selected books. Initially, 30,621 articles were found. Sources covering the years 1970–2021 were used in the research. After the removal of duplicates, 2356 articles were found from digital databases. The inclusion and exclusion criteria for the articles are presented in Table 1.

## 3. Aetiology of MPS

MPS appears to be mainly regulated by psychological and pathophysiological factors, hence, morphological factors [6,7]. Psychosocial factors such as stressful life events, emotional disturbances, psychological distress, and psychiatric disorders (hypervigilance, depression, anxiety, post-traumatic stress disorder, and neurosis) [8,9,10,11,12] contribute to arousal of the central nervous system (CNS). This in turn leads to excessive masticatory muscle activity (MMA) [13]. The masticatory muscles should only be active while chewing and swallowing [14]. However, MMA can result from increased activity in the central nervous system (CNS) triggered by excessive stimuli delivered to the body [15,16,17]. MMA can appear during sleep, which is called sleep bruxism, and/or during wakefulness, which is called awake bruxism [18]. International Consensus from 2018 defined sleep bruxism as a “rhythmic or non-rhythmic muscle activity” and awake bruxism as a “repeated or sustained tooth contact and bracing or thrusting of the mandible” [19]. A new definition points out that both behaviours are neither sleep nor movement disorders in healthy individuals [19]. Functional muscle activity (swallowing and chewing) lasts less than 20 min/day [14], the average bite force is 30.16 kg for a swallow and 26.63 kg for a chew, which totals 7801.8 kg/s per day. In contrast, the average bruxer’s bite force is 54.43 kg (some patients produce up to 113 kg) resulting in 26126.92 kg/s per day [20]. Nevertheless, they can lead to various consequences: flattened/chipped tooth surfaces, microfractures of the tooth enamel, gum recession, loss of teeth, hypersensitivity of teeth to hot/cold/sweet, tongue impaction, ear buzzing/ringing, headache, neck pain, clicking/stiffness/pain in the temporomandibular joints (TMJ), diminished opening, difficulty chewing, sore jaw muscles, changes in facial appearance, and myofascial pain syndrome (MPS, masticatory myalgia) [21,22,23,24,25].

Thus, prolonged exposure to physical and emotional stress can contribute to chronic pain stemming from the TP formation [26]. As much as 75% of patients report that stress is the main reason for a doctor visit [25].

Psychosocial findings are mirrored by Axis II of the DC/TMD protocol which is based on dual-axis diagnosis [2,27]. Axis I assesses a diagnosis with respect to signs and symptoms, and Axis II is used to evaluate psychosocial factors [28]. In Axis II, special attention is given to graded chronic pain, depression, anxiety, somatic symptoms, jaw functional limitations, and oral behaviours [29]. Clinical examination with respect to DC/TMD reveals an important role of the biopsychosocial components in TMD MPS development.

Pathophysiological factors are certain medications (SSRI—selective serotonin reuptake inhibitors, antipsychotics, and psychotropic drugs), CNS disorders, and genetic or familial predisposition [30,31,32]. Other risk factors (potential causes) may be micro- and macro-traumas, overloads, postural disorders, incorrect ergonomics, nutritional deficiencies, infections (e.g., parasitic diseases, babesiosis, Lyme disease, or fungal infections), and sleep deprivation. Furthermore, research has shown that sleep disorders lead to a vicious cycle by increasing pain, which in turn causes further sleep disturbances [33]. Hypothyroidism, low levels of vitamins D, B12, or iron can also play a significant role [34]. Moreover, low-level (sustained or repetitive), eccentric, and concentric (maximal or submaximal) muscle contractions are thought to evoke muscle overload which can lead to the development of TPs [35]. It is worth mentioning that substances connected with increased MMA are nicotine, caffeine, alcohol, and psychoactive substances [36].

The aetiology of TMD also includes biological factors (e.g., sex, hormones), endogenous opioid function, and variations in anatomical genotypes and parafunction [37]. The role of dental occlusion still remains controversial [38]. In turn, adolescents in Class II and III demonstrate a higher prevalence of myofascial pain (1.73 times and 2.53 times) than adolescents Class I [39]. Special attention is attributed to the whiplash injury which is considered as an initiating and aggravating factor of TMD [40]. Currently, the pathophysiology of TMD shifted from mechanical-based theory to a chronic pain biopsychosocial model [40].

### 3.1. MPS and the Limbic System

It is significant to perceive that multiple systems, i.e., the limbic, autonomic, endocrine, somatic, nociceptive, and immune systems, might be affected simultaneously and influence MPS. Understanding the essence of each of the systems may determine the effectiveness of the treatment of myofascial pain syndrome. Due to the involvement of the biopsychosocial component (Axis II of DC/TMD) the apprehension of the physiology and pathophysiology of the limbic system seems desirable [2].

It is a well-known fact that LS is primarily implicated in the adjustment of emotions and consists of several structures including the hippocampus (important for long-term memory, which shapes the perception of events) and the amygdala (integral to the emotional inducement of behaviour and movement). The periaqueductal grey is neurologically associated with the LS and plays a role in defensive conduct and pain modulation [41,42]. The emotional motor system (EMS) relates to a simultaneous combination of outputs from the LS associated with specific emotions, which contributes to a motor response (i.e., masticatory muscles) [23,24,43]. It is hypothesized that mind-brain-body interactions are centrally modulated by EMS and that stressors through efferent pathways generate emotion-particular changes in the body [44,45,46]. Moreover, emotional stress such as anxiety may exacerbate pain through hippocampal mechanisms [47]. In other words, stress not only contributes to muscle tension inducing pain and trigger points (TPs) formation (symptoms of MPS), but it also becomes a physical and emotional stressor causing more stress and pain (hyperalgesia) [47,48]. The outputs from the LS can influence the activity of the autonomic, endocrine, somatic, nociceptive, and immune systems. Therefore, stress can lead to musculoskeletal symptoms such as muscle pain [41,46]. As mentioned, the limbic system has an indirect influence on myofascial pain. The stimulation of trigger points contributes to the expansion of activity in limbic regions [48,49]. This connection between the brain and MPS can be applied in treatment with relaxation techniques (RTs) which have a direct effect on the anterior cingulate cortex—the brain region responsible for pain modulation [50].

### 3.2. MPS and Endocannabinoid System

In the limbic system, the expression of the endocannabinoid system’s (ECS) receptors has been found [51]. The ECS is a crucial component of the musculoskeletal system’s functioning. The ECS helps maintain physiological, emotional, and cognitive homeostasis. The ECS is a biological system that consists of endocannabinoids (neurotransmitters) and cannabinoid receptors (CB1 and CB2) expressed throughout the CNS (including the limbic system), and peripheral nervous system [52]. The CB1 receptors control neurotransmitter release to avoid excessive neuronal activity hence diminishing anxiety, calming, reducing pain, and inflammation [53].

### 3.3. MPS and Autonomic, Endocrine and Immune System

The sympathetic nervous system is responsible for the fight or flight response and shows up as rapid breathing and pulse, and muscle tightening (e.g., masticatory muscles). Therefore, prolonged stress can contribute to MPS [54,55].

In turn, stress activates the hypothalamic–pituitary–adrenal (HPA) axis which has a role in regulating the neuroendocrine response [56,57]. Chronic stress results in an imbalance of the levels of cortisol, growth hormone (the lack of which may show up as fatigue and muscle weakness) [58], testosterone, epinephrine, and norepinephrine (all modulating the pain experience) [17,59]. It may occur as the exhaustion of the system’s raw materials consequently increasing the sensation of pain, an increase in muscle activity, a reduction in repair mechanisms, and an alternation of breathing mechanics [41]. Consequently, a systemic magnesium deficiency may occur and lead to TP formation observed in MPS [41,60]. 

Moreover, persistent stress or depression influences the immune system which predisposes patients to musculoskeletal symptoms [61,62].

## 4. The Occurrence of MPS

MPS is a common disorder; as a general condition, it affects as much as 85% of the general population [63]. Myofascial disorders are the most prevailing causes of chronic headaches and neck pain. Statistics also show that they affect about 50% of people suffering from these symptoms [64]. Moreover, even 100% of patients diagnosed with chronic non-specific neck pain may suffer from MPS [65]. Additionally, neck pain appears with a lifetime prevalence of up to 71% [66,67].

Variability in the prevalence of MPS TMD is due to differences in the methodology, diagnostic tools, and the characteristics of the samples in the various tests. The overall prevalence of myofascial TMD pain amounts to up to 45.3% [68]. It is worth emphasising that individuals with myofascial TMD were significantly more likely to have chronic daily headaches, migraines, and episodic tension-type headaches [69]. Interestingly, 15.4% of Saudi Arabian children suffer from myofascial pain according to the Research Diagnostic Criteria for Temporomandibular Disorders (RDC/TMD). Another 3% demonstrate myofascial pain with limited mouth opening [70]. Myofascial pain with limited mouth opening is reported in 0.2% of Brazilian adolescents, while another 10.3% suffer from myofascial pain [71]. The prevalence of myofascial pain with referral among healthy dental students is 3.7%; the most prevalent diagnosis is myalgia (27.8%) [72]. According to the DC/TMD (Diagnostic Criteria for Temporomandibular Disorders) the prevalence of myofascial pain in a Mexican elderly group is 2.6%, while myofascial pain with limitation in mouth opening is reported in 1.3% [73]. Other research highlights that 18% of people suffer from myofascial pain and 14% report myofascial pain with referral (acc. to the protocol in the DC/TMD) [74]. Among the Ukrainian population, myalgia and myofascial pain syndrome show up in 48.83% of patients (based on the DC/TMD) [75]. Moreover, in the Polish population myalgia occurs in 47.4% and myofascial pain in 14.1% (DC/TMD protocol) [76]. MPS most often performs at the ages between 27.5 and 50, with preference in females and commonly in sedentary individuals [77].

## 5. Diagnosis of MPS

MPS can be diagnosed by palpatory examination, in which TP can be explored. Pressuring on these sensitive areas induces pain and can refer to sensation to different parts of the body through particular patterns. Other symptoms of masticatory myalgia are muscle fatigability, local twitch response, diminished range of motion, muscle stiffness/weakness [13,78,79], and autonomic symptoms such as diaphoresis, lacrimation, flushing, dermatographia, pilomotor activity, and temperature changes [80].

Instrumental analysis may be applied in the course of differential diagnosis. For example, surface electromyography can measure masticatory muscle fatigue [81], and an infrared three-dimensional motion analyser can detect habitual head movements (flexion and extension near the natural head position). It is reasonable to apply an infrared three-dimensional motion analyser, considering that head flexion and extension are always connected with rotation and that the mandibular posture may be influenced by these positions and their muscular determinants [82]. Recent considerations indicated a strong relationship between masticatory muscle dysfunction and upper cervical spine disorders [83].

People suffering from MPS and/or bruxism show an altered biochemical and genetic profile. The main biomarkers reported in patients with bruxism are stress-related such as salivary cortisol and urinary catecholamine; modulators of pain e.g., substance P and genotype and receptor expression [36]. While biomarkers considered in masticatory muscle pain are: algesic (glutamate, serotonin, nerve growth factor, protons, and bradykinin); tissue metabolites (lactate, pyruvate, glucose, and glycerol); and inflammatory mediators (eicosanoids e.g., PGE2, neuropeptides, and cytokines, e.g., IL-1, IL-6, IL-8, TNF) [84].

Biomarkers, ultrasound imaging, magnetic resonance elastography, and electromyography are novel diagnostic tools. However, due to expensive and specialised equipment, they will likely play a limited role in MPS diagnosis [80].

At the moment, the diagnosis of TMD MPS is based on the most widely used DC/TMD protocol.

### Differential Diagnosis with Respect to TMD MPS

It is worth underlining that there are various conditions that should be differentiated from masticatory myalgia. Moreover, some medical disorders (i.e., fibromyalgia, joint dysfunction, and post-traumatic hyperirritability syndrome (PHS)) overlap in the differential diagnosis of MPS (Table 2) [85,86].

It is worth mentioning that according to Axis I and Decision Tree (DC/TMD), TMD MPS should be distinguished from other temporo-mandibular disorders. (Table 3).

## 6. Treatment of MPS

There are two approaches towards the treatment of TMD MPS: directed and self-directed biopsychosocial profile modulation and symptomatic treatment (Table 4).

### 6.1. Directed and Self-Directed Biopsychosocial Profile Modulation

The following section elaborates on the directed, and self-directed biopsychosocial profile modulation because it is the most effective in the treatment and can diminish the risk of TPs development. Notably, behavioural treatment (biofeedback, cognitive-behavioural programs, and relaxation) caused a long-lasting 30–60% decrease in headaches [89]. 

It is worth emphasising that the rationale behind body–mind practice related to the Axis II disorders of DC/TMD is the same neural pathway of the physical aspect of pain and of the emotional aspect of depressive moods [90].

### 6.2. Cognitive Behavioural Therapy

Cognitive behavioural therapy (CBT) is a form of psychotherapy that focuses on how an individual’s feelings and behaviours can be affected by their thoughts, attitudes, and beliefs. Its goal is to change the pattern of cognitions and behaviours that contribute to emotional problems [91,92]. CBT with the inclusion of hypnosis techniques yields a reduction in pain intensity, severity, frequency, and subjective pain index as well as a lower frequency of self-medication, emotional distress, nervous tension, and anxiety. In fact, 70–90% of the participants experienced a significant clinical change, and, additionally, the therapeutic effectiveness persisted after 9 months in 60–80% of the patients [93]. Other research reported a reduction in MPS symptoms in 76.1% of participants that had undergone a CBT course [9]. Moreover, CBT, including bubble breath exercises, was proven to decrease procedural pain and anxiety during buccal infiltration of local anaesthesia in children [94]. 

### 6.3. Biofeedback

Biofeedback (BF) is a technique that regulates physiological activity by the psychological approach. A specific monitoring device is used to observe muscle activity and help patients learn how to keep their muscles relaxed. Bruxers can unlearn their behaviour when a stimulus makes them aware of their adverse muscle activity [7,92]. Several studies showed a correlation between biofeedback usage and a reduction in muscle activity [95,96,97,98,99]. Biofeedback through an in-ear device has the potential to reduce headaches and pain intensity during the night by 50% after three months, and 80% after six months of implementation [100].

### 6.4. Sleep Hygiene Measures

The sleep hygiene measures (SHM) include avoidance of smoking, drinking of alcohol, stimulating drinks, coffee at night, avoidance of eating big meals, decreasing mental or physical activity right before sleep, and good bedroom conditions (light, temperature, fresh air, and a comfortable bed) [30].

### 6.5. Relaxation Techniques

The relaxation technique (RT) is a method that helps individuals with reducing physical and psychological stress and anxiety [101]. Remarkably, 85% of patients undergoing physiotherapy, thermotherapy, and relaxation stopped using any medications. Moreover, a considerable decrease in headache periodicity, intensity, and anxiety was observed [102]. In another 14-month study, headaches, and neck and shoulder pain decreased in 40%, while a drug intake—in c.a. 50% of patients who performed relaxation and postural exercises [103,104]. A meta-analysis of studies between 1997–2007 revealed that RT is effective in reducing anxiety [105].

#### 6.5.1. Meditation

Mindfulness meditation (MM) is a mental training practice that teaches how to focus on the present moment non-judgmentally by paying attention to emotions, thoughts, and sensations [106]. It was shown that meditation increases cerebral blood flow in the regions of the brain which participate in pain experiences and emotional regulation and adjusts the autonomic nervous system (ANS) [50]. Thus, mindfulness meditation is efficient precisely in these zones. Muscle contractibility and MPS are associated with the ANS. MM supported by electroencephalography (EEG) showed short-term and potentially long-term brain state changes. EEG studies proved meditation contributes to a higher amplitude of slower waves such as alpha and theta activity, which can be connected with diminished anxiety and hence, relaxation [107,108]. Transcendental meditation (TM) can decrease muscle sympathetic nerve arousal [109]. Moreover, the physiological indices of stress were lower in individuals practicing TM [110]. It is also known that meditation decreases the level of cortisol—a stress hormone [111]. Additionally, during pain stimulation, the diminishment of pain intensity averaged 27% and the reduction in pain was shown in about 44% of patients during MM [112]. Interestingly, MM training proved to have significant clinical improvements in pain intensity compared with standard care such as pharmaceutical approaches [108].

Furthermore, electroencephalography (EEG) shows decreased alpha and theta activities in the frontal cortex while encountering pain. However, EEG studies proved converse effects such as meditation contributing to a higher amplitude of slower waves (alpha and theta), which is connected with relaxation [107,108]. 

The mindfulness-based stress reduction (MBSR) program is an 8-week course with 45 min of meditation each day. It has a positive effect on pain management. It may contribute to a better mental quality of life and diminish anxiety or depression symptoms in patients with long-lasting chronic pain [113]. 

The Integrated Amrita Meditation (IAM) technique is an integration of yoga (energising exercises), pranayama (short breathing exercise), and meditation. According to Vandana et al. the IAM technique is effective in reducing stress (measured in a group of students aged 18–21). The effectiveness increased after 2 months of follow-up and even more after an 8-month follow-up [114].

#### 6.5.2. Schultz Autogenic Training

Schultz autogenic training (AT) is a psychophysiological self-control method based on a desensitization-relaxation technique and auto-suggestion. AT includes focusing on bodily perception (e.g., heaviness/warmth of arms, legs) which yields relaxation [115,116]. AT diminishes the level of cortisol hence, the effects of stress [117]. It was shown that AT has an influence on the reduction of anxiety and depression [118].

#### 6.5.3. Edmund Jacobson’s Progressive Muscle Relaxation

Edmund Jacobson’s progressive muscle relaxation (PMR) is a therapeutic method induced by deep nerve-muscle relaxation; it is predicated on the assumption that muscle tension is the body’s response to psychological disturbances [119,120]. PMR involves the contraction of a group of different muscles followed by the progressive release of them [121,122]. The pain intensity diminished in all the patients with craniofacial pain (regardless of the symptom/dysfunction level) after 3-months of PMR exercises. Moreover, there was a noticeable reduction in pain frequency, duration, and interference in individuals with psychosocial dysfunction (moderate/high) and adaptive coping (moderate/mild) [123].

#### 6.5.4. Diaphragmatic Breathing

Diaphragmatic breathing (DBT) aims to accumulate tension in the diaphragmatic area. DBT consists of the expansion of the diaphragm so that the lungs can take in more air and it is repeated with the breathing growing slower and deeper [124]. DBT results in better cognitive performance and a reduction in physiological stress (measured by cortisol levels, blood pressure, and respiration), and psychological stress (expressed by the Depression and Anxiety Stress subscale) [125]. In most adults, the sympathovagal stress response ensures physiological balance at 4.5–5.5 breaths per minute. However, the physiological mechanism of this phenomenon remains unknown [126].

#### 6.5.5. Advantages of Relaxation Techniques

Furthermore, there are other applications besides MPS for the previously described techniques. Relaxation techniques are widely used to alleviate stress and psychological and psychiatric disorders. Some of them are related to MPS. In the treatment of nausea due to chemotherapy, cancer palliative care, drug/alcohol dependence, hypertension, menopause, premenstrual syndrome, smoking cessation, and ulcerative colitis RT is used [127]. Moreover, mindfulness meditation is applied in the treatment of skin diseases, immune disorders, and diabetes [128]. Autogenic training is used for patients suffering from asthma, glaucoma, and atopic eczema [127,129]. Biofeedback is being applied in the treatment of asthma, hemiparesis, spasmodic torticollis, blepharospasm, paraparesis, hypertension, cardiac arrhythmias, bowel syndrome, high blood pressure, Raynaud’s disease, hemiplegia, and epilepsy [130,131,132]. Although the data is limited, emerging evidence suggests a possible role for CBT in the treatment of diabetes, cancer, epilepsy, asthma, atopic dermatitis, and HIV/AIDS [133].

### 6.6. Additional Methods

#### 6.6.1. Healthy Lifestyle

Nutrition, hydration, physical activity, rest, and recovery are also essential parts of the well-being of the musculoskeletal system [134,135]. Furthermore, dietary patterns may be an important factor in the therapy of chronic diseases [136]. Interestingly, plant-based dietary patterns reveal pain-relieving effects due to the anti-inflammatory influence. In contrast to higher consumption of protein, fat, and sugar, these fruit and vegetable diets are linked to low levels of inflammatory biomarkers that lead to a decrease in chronic pain and pain intensity [134]. An avocado-soybean unsaponifiable extract appears to have anti-inflammatory effects by inhibiting prostaglandin E2, cyclooxygenase A2, and nitric oxide; and is used to alleviate pain [137,138]. In the case of deficiencies, supplying vitamin C, E, B complex, iron, potassium, and calcium should be considered [139]. Data shows that natural vitamins from fruits and vegetables have clinical advantages over synthetic ones given their availability, nourishment, absorption, and utilization [140,141]. Moreover, acetyl-L-carnitine, alpha-lipoic acid, coenzyme Q10 (CoQ10), and N-acetylcysteine could also play significant roles [142]. Dehydration and diminished electrolyte levels can lead to an impaired function of the musculoskeletal system [135]. Researchers have found that drinks comprising the highest levels of macronutrients and electrolytes (primarily sodium and potassium) promote a higher fluid balance. Orange juice has a higher beverage hydration index than water and it keeps the body hydrated for longer [143].

#### 6.6.2. Cannabidiol Oil

Cannabinoids extracted from cannabis seem to be beneficial in the treatment of pain, inflammation, sleep disorders, and anxiety [144]. Cannabidiol (CBD) oil is one of at least 100 cannabinoid compounds, which is safe and widely used in medicine [145]. CBD has an effect on the CB1 and CB2 receptors, and according to research CBD enhances well-being and reduces inflammation, pain, anxiety, insomnia, and depression [146]. It was shown that 79.2% of all patients had decreased anxiety and 66.7% had improved sleep after one month of CBD treatment. The results persisted for two months after the treatment (78.1% for anxiety and 56.1% for sleep) [147]. A CBD formulation applied onto the masseter muscle reduced its activity, pain intensity, and enhanced its condition in patients with MPS [148]. Moreover, CBD oil used by a 10-year-old child with post-traumatic stress disorder (PTSD) after being sexually abused showed significant relief in anxiety and sleep disturbances. Interestingly, before the CBD therapy, the child was under a standard pharmacological treatment which provided partial relief and significant side effects [53].

#### 6.6.3. Craniosacral Therapy

A promising alternative treatment may be craniosacral therapy which uses gentle fascia palpation techniques to decrease sympathetic arousal by regulating body rhythms and releasing fascial restrictions between the cranium and sacrum [149,150]. This modality improves the body’s function and influences self-regulation by relaxing physical and mental structures [149]. The aim is to reset the human body and restore balance following the body’s imbalance [151].

The craniosacral system has been defined as a semi-closed hydraulic system that releases and absorbs cerebrospinal fluid [152]. This system involves the skull, the cranial sutures, the cerebrospinal fluid, the membranes of the brain, and the spinal cord. It is connected to the musculoskeletal, vascular, endocrine, lymphatic, and respiratory systems as well as to the sympathetic and parasympathetic nervous systems [150,153]. The mobility of the craniosacral system is reflected by the primary respiratory mechanism which manifests as a palpable motion of the cranial bones, sacrum, dura membranes, central nervous system, and cerebrospinal fluid [153].

Craniosacral therapy leads to changes in sensory, motor, cognitive, and emotional processes in the nervous system [152]. Typical is a reduction in muscle tone and an increase in the parasympathetic nervous system response. The characteristics of this therapy are pain relief, an experience of deep relaxation and release, as well as a reduction in anxiety [150]. Consequently, the quality of life and well-being are also improved. In this therapy, a special role is attributed to the C-fibre touch system, social bonding, and interaction which affect the endocannabinoid system [152]. As a result, the release of neuropeptides such as endorphins and oxytocin could be expected [152]. Craniosacral therapy is especially recommended for migraines, headaches, neck pain, TMJ syndrome, bruxism, chronic fatigue, stress and tension-related problems, sensory integration dysfunction, and emotional difficulties [151].

As noted, there are several techniques that provide a consistent reduction in stress and consequently pain relief [154]. Numerous studies show positive effects of relaxation techniques, CBT, and biofeedback on stress and pain [9,93,94,103,104,112,113,123].

## 7. Summary and Future Perspectives

In view of the fact that MPS appears in a majority of the general population, and in many instances it precludes a patient’s normal functioning in society, it is significant to perceive the main cause of MPS. The application of directed and self-directed biopsychosocial profile modulation is believed to bring positive results. Research supports the effectiveness of these methods, which were helpful in diminishing the frequency, intensity, and duration of pain. The common benefit was enhanced well-being. Healthy lifestyle patterns such as rest, physical activity, and nutrition should also be deliberated. Fruit and vegetable dietary patterns alleviate pain by keeping an anti-inflammatory environment and maintaining the highest hydration index because of their macronutrient and electrolyte content. Additionally, CBD oils may also be used in the treatment as they bring back physical, emotional, and cognitive balance alleviating stress and anxiety.

An important role in the treatment of myofascial pain can be educational counselling [155]. Increasing the patient’s awareness and responsibility regarding the psychosocial factors of the disease can be an important treatment tool. Reduction of harmful behaviours and the balance between physiological, psychological, and social factors seem to be a powerful tool to control and alleviate signals and symptoms of TMD [156].

As mentioned above, the treatment of MPS should be deliberated holistically as it is a complex disorder.

## Figures and Tables

**Table 1:** The inclusion and exclusion criteria for articles.

Inclusion Criteria	Exclusion Criteria
Research on myofascial pain syndrome Research on bruxism Research including cognitive behavioural therapy, biofeedback, sleep hygiene measures, relaxation techniques (meditation, Schultz autogenic training, Edmund Jacobson’s progressive muscle relaxation, diaphragmatic breathing), craniosacral therapy MPS research published from 1970	Articles without full text availability Articles in a language other than English Same data that was published at different times

**Table 2:** Medical disorders considered in the differential diagnosis of MPS as a general condition.

**1. Fibromyalgia [80,86]**
**2. Musculoskeletal Diseases:** - temporomandibular joint disorders, - occupational myalgias, - post-traumatic hyperirritability syndrome (PHS), - joint disfunction, - tendonitis and bursitis
**3. Neurological Disorders:** - trigeminal - glossopharyngeal/sphenopalatine neuralgia
**4. Systemic Diseases:** - rheumatoid arthritis, - gout, - psoriatic arthritis, - viral/bacterial/protozoan infections
**5. Heterotopic Pain of Central Origin**
**6. Axis II-Type Personality Disorders** - psychogenic pain, - painful behaviours
**7. Drug Reactions**

**Table 3:** Disorders diagnosed according to Axis I and the Decision Tree (DC/TMD) in the differential diagnosis of TMD MPS.

**I. Pain-Related TMD and Headache** A. myalgia B. myalgia Subtypes: - local Myalgia - myofascial Pain - myofascial pain with spreading - myofascial pain with referral C. Arthralgia D. Headache attributed to TMD
**II. Intra-Articular Joint Disorders** Disc displacement with reduction Disc displacement with reduction with intermittent locking Disc displacement without reduction with limited opening Disc displacement without reduction without limited opening
**III. Degenerative Joint Disorder** **IV. Subluxation**

**Table 4:** MPS treatment [7,87,88].

Directed and Self-Directed Biopsychosocial Profile Modulation	Symptomatic Treatment	Additional Methods
1. cognitive behavioral therapy (CBT), 2. biofeedback (BF), 3. sleep hygiene measures, 4. relaxation techniques (RT) (a) mindfulness meditation (MM), (b) mindfulness-based stress reduction (MBSR) program (c) Integrated Amrita Meditation (IAM) (d) autogenic training (AT), (e) progressive muscle relaxation (PMR), (f) diaphragmatic breathing technique (DBT)	1. occlusal splints, 2. pharmacological therapy, 3. physiotherapy, 4. occlusal equilibration, 5. prosthodontic reconstruction, 6. acupuncture, 7. botulinum toxin, 8. transcutaneous electrical neuromuscular stimulation (TENS), 9. contingent electrical stimulation [7,87,88]	1. plant-based diet/ fruits and vegetable diet 2. hydration 3. physical activity 4. rest 5. cannabidiol oil 6. craniosacral therapy

## Data Availability

Data sharing not applicable.
